# Hospital Meetings, &c.

**Published:** 1897-03-06

**Authors:** 


### The Institutional Workshop.

### HOSPITAL MEETINGS, &C.

### METROPOLITAN CONVALESCENT INSTITUTION.

The fifty-sixth annual general meeting of the governors of the Metropolitan Convalescent Institution was held on Tuesday, 23rd ult., at the office, 32, Sackville Street, W., Mr. Richard B. Wade in the chair. Amongst those present were Sir John Tilley, K.C.B., the Rev. J. Neal, Mr. Samuel Osborn, Mr. S. Royson, and Mr. C. Holmes. The report of the Board of Management stated that during the past year 5,064 men, women, and children were admitted to the homes, situated at Walton-on-Thames, Bexhill, and Broadstairs, nearly all of whom left after a residence of about three weeks either quite well or much improved in health. The new home for children at Broadstairs, which contains 100 beds with special wards for surgical cases still under treatment, was opened in July last, and between that date and the end of the year 618 children were received. The total cost of the building and the furnishing was £15,064, which exceeded by £1,941 the funds at the disposal of the Board, and a very earnest appeal was made for donations to make up the deficiency. The report and statements of account having been adopted, the thanks of the governors were accorded to the hon. medical officers and the auditors for their services during the past year, and a vote of thanks to the chairman brought the proceedings to a close.

### KING'S COLLEGE HOSPITAL.

The annual court of governors of King's College Hospital was held in the board-room on Wednesday, the 24th ult., Mr. Richard Twining in the chair.

The report, which was adopted on the motion of Mr. Theodore Carter, seconded by Mr. A. H. Bailey, stated that the number of in-patients treated in 1896 was 2,703, as against 2,717 in 1895; whilst the out-patients amounted to 25,772, against 26,875 in the previous year. The committee stated, in reference to recent discussion of the subject, that they did not believe that there was any appreciable misuse of the benefits of the out-patient department of the hospital. For many years past all applicants for treatment had been seen and registered by a registrar specially appointed for this purpose, and members of the committee had themselves attended in the rooms where the out-patients were seen. The result of this continuous observation led them to think that there was no such misuse of the charity as was sometimes apprehended. The out-patients also were not more in number than could be satisfactorily treated by the staff. The supply of necessary clothing for the poorer patients in the hospital had now been provided for by the establishment of a Hospital Guild in co-operation with the sisters of the wards. The total income (including £3,860 legacies) amounted to £17,634, and the expenditure (including £578 spent on building improvements) to £18,434.

The election of the committee of management and the various officers completed the business of the meeting.

### EAST LONDON HOSPITAL FOR CHILDREN.

The annual meeting of the East London Hospital for Children was held in the board-room of the hospital on Wednesday, 24th ult., the Earl of Strafford in the chair. The report stated that the purchase of the site at Bognor for the convalescent home had been completed, and the plans for the home had been settled, accommodation being provided for 28 patients. The ordinary receipts had amounted to £5,269, legacies to £1,663, and the festival dinner to £1,315. The ordinary expenditure was £7,387, and the expenditure on improvements £118. 1,496 in and 34,941 out patients were treated during the year. The report having been adopted, the Bishop of London was, on the motion of the Chairman, elected a patron of the institution. The committees and honorary officers were re-elected, and the trustees were authorised to sell or let on building lease the site of the old hospital buildings. A vote of thanks to the chairman for presiding completed the business of the meeting.

### ST. JOHN'S HOSPITAL FOR DISEASES OF THE SKIN.

The annual board of governors was held on Wednesday, the 24th ult., at the Hotel Victoria, Northumberland Avenue, S.W., Mr. G. A. Berkeley occupying the chair, in the absence, through illness, of the Earl of Chesterfield, who was to have presided.

The report was adopted subject to the addition of a reference to the services of Mr. Milton, the founder of the hospital. The election of the honorary officers and the board of management, and the usual votes of thanks, brought the meeting to a close.

### CANCER HOSPITAL.

The forty-sixth annual meeting of the governors of this charity was held in the board-room of the hospital, Brompton, S.W., on Wednesday, the 24th ult., Sir George Samuel Measom occupying the chair. From the report of the committee, which was read by the secretary, it appears that during the past year 829 in-patients and 1,516 out-patients were received, whilst the total number of attendances of out-patients was 13,786, these figures being in excess of any previous year since the hospital was founded. The report and balance-sheet were adopted, and the usual votes of thanks accorded, after which the proceedings terminated.

### MIDDLESEX HOSPITAL.

At the quarterly general court of the governors of the Middlesex Hospital held on Thursday, 25th ult., Mr. Henry N. Custance in the chair, the report for the year 1896 was adopted. The number of patients treated during that year was 3,598 in, and 44,484 out patients, an increase in both departments over 1895, due not to the abuse of the out-patient department, but to the situation of the hospital in the midst of a dense population having little means of coping on their own account with sickness and distress, and exposed by the nature of their occupation and surroundings to many risks of accident. The chief features of the year had been the opening of the Convalescent Home at Clacton-on-Sea, and the amalgamation of the medical school with the hospital. The amalgamation scheme in the latter case provides for the taking over by the hospital of all the property of the school, together with its liabilities, and henceforth the hospital will receive the gross income of the school, and, after payment of the necessary charges, will provide, according to the profits obtained, a fixed annual sum for the remuneration of the teaching staff.

Sir Ralph Thompson, K.C.B. (Chairman of the Weekly Board), proposed the adoption of the report. So far as could be seen the amalgamation of the school with the hospital promised to be most successful. The Board was now considering the question of alterations and additions to the school buildings. Speaking generally, the record of the hospital during the past year showed a steady increase in prosperity and efficiency and in the esteem in which it was held by the public.

Lieut.-Colonel Mears, in seconding, said that two items in the report filled the Board with anxiety, the debt of £29,000 on the new Convalescent Home, and the £6,000 still to be collected for the new cancer wing.

The adoption of the report was carried unanimously.

The retiring members of the Weekly Board and the annual officers were re-elected.

The Trustees were empowered to sell sufficient stock to realise £10,000 for the purpose of (1) advancing £5,000 to the Samaritan Fund; (2) repaying a loan by the bankers; and (3) meeting current expenses.

The title of Physician to the Out-patients was conferred on Dr. C. J. Biss, the senior assistant physician.

The meeting then terminated.

### GREAT NORTHERN CENTRAL HOSPITAL.

The annual general meeting of the governors was held in the board-room of the hospital on Friday, 26th ult., Mr. C. T. Murdoch, Chairman of the Committee of Management, presiding.

The Chairman, in moving the adoption of the report, said that during the year 1896 there had been 107 beds available for patients in the wards, against 55 in 1895. The fact that these 107 beds had been occupied almost continuously (the daily average number of beds occupied being 99), while many important cases had had to be refused, showed that the time had arrived when an earnest endeavour should be made to use the two wards still unopened. There had been an increase in the out-patients of nearly 3,000 when compared with 1895. The system pursued by the hospital for some years past had been to appoint a particular officer whose duty it was to enquire into the financial condition of those who sought relief. In addition to that a special committee had been appointed to enquire into the matter, and from their report it appeared that during 1895 inquiry was deemed necessary in about 47 per cent. of the cases, and that of the whole number of out-patients less than 2½ per cent. were found to be unsuitable. After dealing with the various items in the report, he referred to the death of Dr. William Cholmeley, one of the founders of the hospital, which had taken place during the year.

Mr. Austin seconded, and the report was adopted.

Votes of thanks were passed to the several committees connected with the hospital, and the retiring honorary officers were reappointed.

The proposed alterations in the rules were not placed before the meeting, as sufficient notice of the suggested amendments had not been given to the governors.

A vote of thanks to the Chairman concluded the proceedings.

### SEAMEN'S HOSPITAL.

The annual court of the Seamen's Hospital Society was held at the Royal United Service Institution, Whitehall, S.W., on Thursday, 25th ult., Earl Spencer, K.G., P.C., in the chair. Those present included: Admiral H. de Kantzow, Admiral Sir Anthony H. Hoskins, G.C.B., Admiral Sir W. J. Hunt-Grubbe, K.C.B., Admiral E. S. Adeane, C.M.G., Admiral H. Boys, His Excellency M. Takaaki Kato (Japanese Minister), the Naval Attaché to the Italian Embassy, Sir Frederick Young, K.C.M.G., Major the Hon. W. Rowley, Captain C. A. White, and Captain Francis M. Ommanney.

The Chairman, in opening the proceedings, said he felt sure every patriotic Englishman ought to do what he could for an institution which accomplished so much for those who served his country—for those men who did so much to promote that which had the greatest influence on the prosperity of this empire. Not only, too, did the society confer enormous benefits on the seamen of the United Kingdom, but it extended those benefits to the seamen of the world. The financial position of the society was not, however, in a wholly satisfactory state. The work done since the year 1890 had increased by about 7,000 patients, but the income had not increased in proportion, so that while prior to that year the income had exceeded the expenditure, since then the reverse had, with but a slight exception in 1895, been the case. He had calculated that if each ship belonging to the Empire contributed half-a-farthing on her tonnage an annual sum of nearly £7,000 could be raised.

The Japanese Minister moved the adoption of the report and the election of the officers and committee of management, and in doing so said that there were always a numbe of Japanese sailors coming to the port of London, and that number would be considerably increased in the future. H announced that in order to show the appreciation of his Government in the work of the hospital the new Japanese battleship, the "Fuji," which was being built on the Thames and which was now rapidly approaching completion, would be exhibited in aid of the funds of the society either next month or the month after.

Admiral Boys seconded, and the motion was carried.

The Naval Attaché to the Italian Embassy moved, and Sir Frederick Young seconded, a vote of thanks to the contributors to the funds of the society, which was carried.

Mr. Percival A. Nairne, in proposing a vote of thanks to the chairman, said that what pressed most upon the society was the inadequacy of the branch hospital at the Docks to cope with the urgent cases brought to it. The percentage of very severe cases brought to it was, he felt assured, greater than the percentage of similar cases at any other hospital in London or, indeed, in the kingdom. Its enlargement, which they were now contemplating, would entail a considerable increase for its annual maintenance, and he hoped the public would respond liberally to their appeal.

Admiral Sir W. Hunt-Grubbe seconded, and the motion was carried unanimously.

The Chairman responded, and the meeting terminated.

### CENTRAL LONDON OPHTHALMIC HOSPITAL.

The annual meeting of the general council of governors of the Central London Ophthalmic Hospital was held at the hospital on Thursday, 25th ult., Mr. J. Stamford Burrows presiding.

On the motion of Colonel W. J. Tatham, seconded by Mr. Henry Clarke, the report was adopted. This report stated that the number of patients in 1896 exceeded all previous records, being 10,720, of whom 197 were treated in the wards. The ordinary income had amounted to £1,171, and the expenditure to £1,175, of which £41 was placed to the account of extraordinary repairs.

The Chairman pointed out that the work of the hospital had so increased that they had outgrown their accommodation. About £15,000 was required for the enlargement of the hospital, towards which £2,046 had been collected.

The reappointment of the honorary officers and the usual votes of thanks completed the business.

Mr. T. Brittin Archer, in acknowledging the vote of thanks to the surgical staff, referred to the agitation on behalf of the Central Hospitals Board for London. Two forces were behind that agitation, the General Practitioners and the Charity Organisation Society. The former were extremely reluctant that anybody should go into the out-patient rooms with even a very small sum of money in their pockets, and the latter would like to have the distribution of the charitable bequests of London in their own hands. The patients who attended at that hospital were of two classes, those who were absolutely destitute, and those who, earning say £1 or £2 a week, after paying their rent and for their food, and dressing respectably as they had to do, had but little left to pay a man who was capable of treating eye diseases. The latter class paid the hospital 2s. 6d. for a monthly ticket, or about 4½d. an attendance. Which was the better, that these indigent people should come to that hospital and be properly treated for that small sum (thereby helping the committee to treat for nothing those who had no money), or that they should go to the general practitioner who had no facilities for, nor knowledge of treating ophthalmic cases? As to the Charity Organisation Society, he was not enthusiastic about their capability for reforming anything, even their own machinery.

